# Self-regulation of healthy nutrition: automatic and controlled processes

**DOI:** 10.1186/s40359-016-0108-5

**Published:** 2016-01-30

**Authors:** Heike Eschenbeck, Uwe Heim-Dreger, Amina Steinhilber, Carl-Walter Kohlmann

**Affiliations:** Department of Psychology, University of Education Schwäbisch Gmünd, Oberbettringer Str. 200, D-73525, Schwäbisch Gmünd, Germany

**Keywords:** Eating behaviour, Self-regulation, Controlled behavioural processes, Automatic behavioural processes, Implicit association test

## Abstract

**Background:**

Self-regulatory behaviour refers to both controlled and automatic processes. When people are distracted, automatic over controlled processes prevail. This was analysed with regard to nutritional behaviour (food choices, beverage intake) in situations of low or high distraction.

**Methods:**

A self-concept Implicit Association Test (IAT) was adapted to assess the implicit associations of self (vs. other) with healthy (vs. unhealthy) food. Explicit preferences for healthy and unhealthy food and the diet’s healthiness were measured by self-report. Both implicit and explicit measures were used as predictors of nutritional behaviour. Among 90 undergraduates, the choice of fruit versus snack in a food choice task (low distraction) and the amount of mineral water and soft drinks consumed in a taste comparison task to cover liquid intake (high distraction) were observed.

**Results:**

In the low distraction situation, food choice was predicted solely by explicit measures. Fruits were chosen less, when unhealthy foods were explicitly liked. In the high distraction situation, mineral water intake was predicted solely by the IAT. Participants implicitly associating themselves with healthy foods drank more mineral water than those implicitly associating themselves with unhealthy foods.

**Conclusions:**

Nutritional behaviour is influenced by both automatic and controlled processes depending on the available capacity for self-regulation.

## Background

Explicit, or controlled, and implicit, or automatic processes are seen as basic determinants of human behaviour (e.g., [1, 2]). These dual behavioural processes also play a central role in health behaviours, such as eating (e.g., [3]; for a review, see [4]). According to Fazio [1], on the one hand, behaviour can be based primarily on a deliberate and conscious analysis of its costs and benefits. On the other hand, when an individual has, for example, little opportunity to engage in controlled processing, behaviour may be driven automatically. Automatic processing is fast, requires little effort, is difficult to control and can operate in situations with high workload (see [5, 6]). One explanation for why some people act more controlled than others refers to self-regulation, which is needed to resist engaging in problematic behaviour, such as eating fatty or forbidden foods ([7]; see [8]). Self-regulatory behaviour facilitates pursuing long-term goals and meeting social expectations. However, if a person’s self-regulation is impaired due to increased cognitive load such as distraction, the automatic process prevails over the controlled, and the person gives in to impulses triggered by tempting stimuli. As a consequence, one might consume palatable food such as chocolate, ice cream or crisps despite the intention to eat healthy (e.g., [9]; see also [10] for beneficial effects of cognitive load for self-regulation via reduced attention to temptation).

Whereas explicit attitudes and intentions (e.g., a preference for a healthy diet) as determinants of controlled processes can be assessed by direct measures such as self-reports, implicit attitudes and self-concepts as determinants of automatic processes can be assessed indirectly by measures of association strength. A prominent implicit measurement tool is the Implicit Association Test (IAT, [11]; see also [12]). The IAT measures the strength of automatic associations between concepts by comparing the response times on two combined discrimination tasks. It has been used to assess implicit attitudes, stereotypes or self-concepts [12–14] related to a variety of domains (e.g., consumer preferences, alcohol and drug use; see [15]).

In health-related domains, especially attitude IATs have been adapted to test issues such as smoking behaviour, alcohol consumption, and food and nutrition (e.g., [16–19]). Maison et al. [20] conducted a study in the context of marketing and consumer research. The target concepts were high-calorie (e.g., chocolate, nuts) and low-calorie (e.g., fruits, lettuce) foods, and the attribute categories were pleasant and unpleasant words. The results showed that the reaction times were shorter when the category “low-calorie foods” was paired with pleasant words than when the category “high-calorie foods” was paired with pleasant words. These results indicate that the participants had more positive implicit attitudes towards low-calorie than high-calorie foods.

A number of studies have been conducted to test the predictive power of implicit attitudes (assessed by the IAT) and explicit attitudes (assessed by self-reports) towards specific behavioural food choices (e.g., [18, 21, 22]). The main assumption of most of these studies was that implicit attitudes are better able to predict criterion variables (e.g., food choices) than explicit attitudes and food preferences. For example, a study by Richetin et al. [23] showed that an IAT on attitudes towards fruits (e.g., fruit, apple, banana) versus snacks (e.g., snacks, candy, chocolate) had both predictive and incremental validity for the choice between the two. Thus, the participants who had an implicit positive attitude towards fruit chose fruit more frequently than a snack. The IAT score also remained a significant predictor of behavioural choice alongside explicit attitudes. However, findings are not consistent. In a study by Karpinski and Hilton [22] (Study 2), an IAT on attitudes towards healthy versus unhealthy food (using the concepts of “apples” and “candy bars”) failed to predict the choice between an apple and a candy bar, but an explicit attitude measure was able to predict it. Experimental procedures (i.e., tasks) and IAT measures require some attention for the observed inconsistencies.

Based on dual-process models (e.g., [1]), IAT measures are expected to be especially able to predict spontaneous and less controlled behaviour during tasks that absorb attention. In these situations, self-regulation may shift from controlled to more automatic processes. In a series of three studies, Friese et al. [21] manipulated control resources with additional tasks (e.g., digit span, emotion suppression) and demonstrated a moderated predictive validity for implicit and explicit measures across different behavioural domains (e.g., the choice behaviour of fruit versus chocolate, crisps consumption). The IAT predicted choice and consumption behaviour in the high cognitive load condition, but not in the low cognitive load condition. For explicit attitude measures, the opposite was true. Therefore, the mode of self-regulation is affected by available cognitive capacity [17]. To systematically evaluate the role of implicit, automatic and explicit, controlled behavioural processes in the self-regulation of healthy nutrition, at least two aspects will be considered. First, implicit and explicit measures of behavioural tendencies must be assessed, and second, health-related behaviour (e.g., food choice or food and beverage consumption) must be observed in at least two situations that vary in the degree of attention absorption and therefore in the distraction from healthy nutrition.

In nutrition research with the IAT, previous research has mostly relied on attitude IATs that combine a (more or less general) concept classification (e.g., healthy vs. unhealthy food items) with an attribute classification representing positive versus negative valence (e.g., pleasant vs. unpleasant). An alternative may be self-concept IATs that combine the contrast of self versus other with a nominal contrast (e.g., healthy vs. unhealthy food items). According to Olson and Fazio [24], the standard attitude IAT can be contaminated by associations that do not contribute to one’s evaluation of an attitude object. They argue that the labels “pleasant” and “unpleasant” carry specifically normative implications. That is, something about the item (e.g., food item) makes it “pleasant” or “unpleasant”, not something about the participant’s attitude towards it. This appears to be problematic for items that are typically portrayed as normatively either positive or negative. This may also be the case for healthy and unhealthy food items. Alternatives may be “personalised” IATs (with the labels “I like” and “I don’t like”; see [24]) or self-concept IATs (with “self” vs. “others” as target concepts; see [25]) that can be used to assess association strengths with attribute categories (e.g., healthy vs. unhealthy foods).

In the present study, a self-concept IAT was used as an implicit measure of automatic processes in addition to explicit measures of controlled processes to predict health-related nutritional behaviour in two situations that varied in the degree of attention absorption (i.e., distraction from health-related aspects of nutrition). A food choice task of fruits versus snacks (low distraction) and a task on comparing the tastes of water and soft drinks (high distraction) were administered. The food chosen in the low distraction task and the amount of mineral water and soft drinks consumed in the high distraction task served as the behavioural measures to be predicted. Thus, as an extension of previous studies, in the present study distraction was an integral part of the task itself to absorb attention and impair controlled processing. Moreover, Friese et al. [21], p. 405 discussed the possibility that the exertion of control may be harder for continuous consumption behaviours than for single-act food choices. It was hypothesised that the food choice task would cover the more controlled aspects of self-regulation and that therefore, food choice would be better predicted by explicit measures. We further expected that the consumption of mineral water and soft drinks in the taste comparison task would cover more automatic aspects of self-regulation with regard to nutrition because of the attention absorption and the distraction from healthy nutrition. Therefore, in the latter situation, the IAT was expected to predict beverage intake.

## Methods

### Participants

A total of 90 undergraduate students (79 women and 11 men) were recruited on campus during the orientation days of the semester. Their ages ranged between 19 and 38 years (*M* = 21.56, *SD* = 3.83). Their body mass index (BMI, based on self-reported weight and height) ranged between 17.2 and 35.9 kg/m^2^ (*M* = 21.9, *SD* = 3.5, *n* = 88). Eleven percent of the sample were underweight (i.e. definition of BMI < 18.5 kg/m^2^), 76 % were of normal weight (BMI ≥ 18.5 and < 25 kg/m^2^), 9 % were overweight (BMI ≥ 25 and < 30 kg/m^2^), and 3 % were obese (BMI ≥ 30 kg/m^2^).

### Procedure

The first step in the study was a Nutrition IAT. Subsequently, a questionnaire on the healthiness of the students’ diets and their preferences for certain foods was distributed to the participants and to be returned within the next three days. After the Nutrition IAT, the subjects went to another room, where a second experimenter presented four measured cups of drinks and a liquid taste evaluation questionnaire. The taste task served as a distraction from the health-related aspects of the beverages (high distraction situation). Finally, a behavioural food choice between fruits and snacks was presented to the participants. In this task, participants were shielded from possible distractions (low distraction situation).

The study was conducted according to the ethical guidelines of the German Psychological Society and of the Declaration of Helsinki. The project was approved by the Research Ethics Committee of the University of Education Schwäbisch Gmünd. On the monitor, prior to starting the Nutrition IAT, participants were informed that beginning the task would be taken to indicate consent to participate.

### Measures

#### Self-report measures

The participants indicated the healthiness of their diets with one item (healthy diet^1^, i.e., “I eat a balanced diet: a lot of fruit, vegetables, and whole grains, limited consumption of fats, sugars, and cholesterol”, [26]), and responses ranged from *not at all* = 1 to *very much* = 4. In addition, participants rated their preferences for six unhealthy and six healthy foods (i.e., “Please indicate on a scale from 1 = *not at all* to 4 = *very much* the extent to which you like the following food.”). Food items were taken from the Nutrition IAT described below. The internal consistency for the preference for healthy food was *α* = .69, and that for the preference for unhealthy food was *α* = .52.

### Nutrition IAT

The present study’s Nutrition IAT integrated a self-concept measure using the categories of self versus other (see [25]) rather than pleasant versus unpleasant, the categories of attitude IATs (e.g., [23]). The stimuli for the target concept *self* versus *others* were a series of words; the category “self” corresponded to I, self, my, me, and own, and the category “others” corresponded to they, them, your, you, and others (translated from German; see [27]). The stimuli for the attribute category were photographs of *healthy food* items (i.e., apple, banana, carrot, coarse whole meal bread, pepper, and salad) and *unhealthy food* items (i.e., chocolate, chips, crisps, ice cream, gummy bears, and pizza).

The Nutrition IAT was administered on a laptop computer with the programme Inquisit 3.0.4 (Millisecond Software, Seattle, USA). The IAT procedure consisted of seven blocks [11]. In the first block, the participants practiced target concept discrimination by categorising stimuli as self or other (20 trials). In the second block, they did the same for attribute discrimination by sorting photographs of food articles into the categories healthy and unhealthy (20 trials). The first series of critical trials consisted of 20 practice trials (Block 3) and 60 critical trials (Block 4). The participants categorised items into two combined categories, each of which assigned an attribute and a target concept to the same key (e.g., self + unhealthy for the left key, other + healthy for the right key). In Block 5, the participants practiced the switched key assignment for the attribute stimuli (20 trials). Block 6 and Block 7 (the second critical block) were complementary to Block 3 and Block 4; the only difference was that these blocks included the reversed key assignment that the participants had practiced in the previous block (e.g., self + healthy for the left key, other + unhealthy for the right key). The IAT score was calculated using the improved scoring algorithm proposed by Greenwald et al. [28]. The IAT score is based on individualized differences between mean reaction time for self + unhealthy food and mean reaction time for self + healthy food. Positive scores indicate a stronger association between the self and healthy food than between the self and unhealthy food. No participants’ error rates exceeded 20 % (average error rate: 3.46 %), and no mean latencies exceeded two seconds. Applying the scoring algorithm (see [28]) to two mutually exclusive subsets of the IAT combined-task trials, the reliability (split-half method) of the IAT score was *r* = .86.

### Behavioural measures

Food choice (low distraction situation): At the end of the session, the participants were offered a selection of fruits (i.e., apples, bananas) and snacks (i.e., small chocolate bars, sachets with gummy bears), in each case approximately hand-sized. They were asked to choose something (one piece) as a small reward for their participation. Their choices were coded as 1 = fruit and 0 = snack. The choice of fruit was regarded as healthy behaviour, whereas the choice of a snack was regarded as unhealthy behaviour. Three female participants did not take either fruit or a snack. Thus, all analyses relating to the food choice were calculated without the data of these three participants.

Liquid intake in the taste comparison task (high distraction situation): Four transparent cups, each filled with 155 g of drink (i.e., mineral water and three types of soft drinks: coke, sparkling apple juice, lemonade), were used for the taste comparison task to cover liquid intake. The participants were instructed to taste each of the four drinks and to rate their tastes to absorb their attention. They were given 9 min. A liquid taste evaluation questionnaire with four adjectives (i.e., fresh, tangy, natural, and sweet) and an overall taste assessment (i.e., “Please indicate on a scale from 1 = *not at all* to 6 = *very much* the extent to which you like the drink.”) was used. After the test, the liquid left in each cup was taken to another room and weighed by another experimenter. Thus, for the participants, the taste rating was the task. They were uninformed that the liquid left in each cup was being used to calculate their intake of each beverage.

### Statistical procedure

The associations between the variables were analysed using zero-order correlations. In addition, we applied regression analyses using hierarchical entry procedures (forced entry) to evaluate the extent to which gender and age (variable Block 1), the three self-report measures healthy diet, preference for healthy food, and preference for unhealthy food (variable Block 2), and the Nutrition IAT (variable Block 3) contributed to the variance in each of the behavioural measures. Final equations were defined by a significant (*p* < .05) increase in *R*^*2*^ change after the last block was entered.

## Results

### Initial analyses

#### Self-report measures

Preferences for healthy food and unhealthy food with food items taken from the Nutrition IAT were analysed by a MANOVA with category (healthy food, unhealthy food) as the within-subject factor and gender and age as control factors. No significant effect for category was obtained, Wilks-Lambda = .99, *F* (1, 87) = 0.70, *p* = .41, Eta^2^ = .008. Therefore, healthy and unhealthy IAT stimuli are assumed not to mainly reflect positive (i.e., liked) versus negative (i.e., unliked) stimuli for the participants.

A self-reported healthy diet was associated positively with the preference for healthy food (*r* = .32) and negatively with the preference for unhealthy food (*r* = −.25). There was no statistically significant relationship between the preferences for healthy and unhealthy food. With the exception of one positive correlation of *r* = .41 between healthy diet and age, the self-report measures did not have statistically significant associations with gender or age (see Table 1).

**Table 1:** Means, standard deviations, and correlations among the variables

Variables	*M*	*SD*	1	2	3	4	5	6	7	8	9
Socio-demographics											
1. Gender ^a^	88 % female										
2. Age	21.56	3.83	−.07								
Self-report measures											
3. Healthy diet	2.69	0.74	.07	.41***							
4. Preference for healthy food	20.06	2.74	.19^+^	.10	.32**						
5. Preference for unhealthy food	18.98	3.26	.10	−.08	−.25*	−.10					
Implicit measure											
6. Nutrition IAT ^b^	0.41	0.40	−.09	.18^+^	.17^+^	.03	−.11				
Behavioural measures											
7. Food choice ^c^	0.38	0.49	−.06	.09	.26*	.25*	−.34**	.11			
8. Mineral water intake (g)	49.83	42.05	−.27*	.28**	.20^+^	.07	−.24*	.30**	.08		
9. Soft drink intake (g)	38.45	31.58	−.44^***^	.13	.05	−.02	−.01	.10	−.01	.64***	
10. Difference: Mineral water intake − soft drink intake (g)	11.38	32.75	.08	.23*	.21*	.11	−.31**	.30**	.11	.67***	−.15

*Note. N* = 90. ^a^1 = female, 0 = male. ^b^High scores indicate a stronger association between self + healthy food than between self + unhealthy food. ^c^ *N* = 87. 1 = fruit. 0 = snack

^+^ *p* < .10, * *p* < .05, ** *p* < .01, *** *p* < .001 (two-tailed)

#### Nutrition IAT

The IAT effect differed significantly from zero: *M* = 0.41, *SD* = 0.40, *t* (89) = 9.85, *p* < .001. This result indicates that the participants associated themselves more with healthy than with unhealthy food items. The IAT effect did not show significant associations with gender or age (see Table 1).

#### Behavioural measures

In the food choice task, the participants chose fruits (38 %) less frequently than snacks (62 %). Our analyses revealed no significant effects for gender or age.

Mineral water intake ranged between 0 and 152.9 g, with a mean of 49.8 g, and the intake of each of the soft drinks varied between 0.6 and 152.1 g, with a mean intake of 38.5 g averaged across the three soft drinks. We obtained a general score of preference for mineral water over soft drinks by subtracting the mean soft drink intake from that of mineral water (range from −85.07 to 134.17). The mineral water intake was positively associated with the soft drink intake (*r* = .64, after controlling for gender: *r*_p_ = .46, *p* < .001). With regard to gender and age, the results show that women generally drank less mineral water (*r* = −.27) and soft drinks (*r* = −.44) than men. The older participants drank more mineral water than the younger participants (*r* = .28).

The correlation coefficients of food choice with mineral water and soft drink intake and the difference between mineral water and soft drink intake were not statistically significant (range of *r*s: −.01 to .11, see Table 1).

### Correlations of the self-report measures and the Nutrition IAT with behavioural measures

In the food choice task (low distraction situation), the associations between the three self-report (i.e., explicit) measures of healthy diet and preferences for healthy and unhealthy food with food choice were statistically significant. Participants who chose fruit instead of a snack reported healthier diets (*r* = .26). In addition, they reported liking healthy food (*r* = .25) and disliking unhealthy food (*r* = −.34). Finally, there was no statistically significant relationship between food choice and the Nutrition IAT (*r* = .11).

In the taste comparison task (high distraction situation), however, the mineral water intake and the difference between mineral water and soft drink intake were related to the Nutrition IAT (*r*s = .30). Participants who drank absolutely or relatively more mineral water in the taste comparison task implicitly associated themselves more with healthy than with unhealthy food items. Furthermore, the self-report (i.e., explicit) measures of healthy diet (*r* = .20 and *r* = .21) and preference for unhealthy food (*r* = −.24 and *r* = −.31) were associated with mineral water intake. For soft drink intake, associations with explicit and implicit measures could not be registered.

### Regression analyses

With regard to food choice in the low distraction situation (i.e., the food choice task), only step 2, with the self-report (i.e., explicit) measures, was significant; *ΔR*^*2*^ = .19, final equation: *F* (5, 86) = 3.71, *p* < .01 (see Table 2). The only significant predictor was the explicit preference for unhealthy food (*β* = −.28). Participants with a preference for unhealthy food tended not to choose fruit instead of a snack.

**Table 2:** Hierarchical regression analyses predicting food choice and beverage intake

Step	Predictor	Food choice ^c^		Mineral water intake		Soft drink intake		Difference: mineral water intake − soft drink intake	
		*ΔR* ^*2*^	*β*	*ΔR* ^*2*^	*β*	*ΔR* ^*2*^	*β*	*ΔR* ^*2*^	*β*
1	Gender ^a^	.01	−.08	.14**	−.24*	.20***	−.43***	.06^+^	.13
	Age		−.02		.19^+^		.10		.17
2	Healthy diet	.19**	.15	.05	.04	.01		.09*	.01
	Preference for healthy food		.20^+^		.07				.03
	Preference for unhealthy food		−.28**		−.16				−.28**
3	Nutrition IAT ^b^	.00		.05*	.22*	.00		.06*	.24*
Total *R* ^2^		.19**		.23**		.20***		.21**	
*N*		87		90		90		90	

*Note*. ^a^1 = female, 0 = male. ^b^High scores indicate a stronger association between self + healthy food than between self + unhealthy food. ^c^1 = fruit, 0 = snack

Total *R* ^*2*^ and *β* coefficients are for the final equation of the regression analyses

^+^ *p* < .10, * *p* < .05, ** *p* < .01, *** *p* < .001

During the taste comparison task (high distraction situation), the mineral water intake and the difference between mineral water and soft drink intake were predicted by the Nutrition IAT after the other variables were controlled for (see Table 2); final equations: mineral water intake: *F* (6, 89) = 4.18, *p* = .001; soft drink intake: *F* (2, 89) = 11.09, *p* < .001; difference between mineral water intake and soft drink intake: *F* (6, 89) = 3.65, *p* < .01. Specifically, the significant predictors for mineral water intake were gender (*β* = −.24) and the Nutrition IAT (*β* = .22). The only predictor for soft drink consumption was gender (*β* = −.43). In general, women drank less mineral water and soft drinks than men. The statistically significant predictors for the difference between mineral water and soft drink intake were the explicit preference for unhealthy food (*β* = −.28) and the Nutrition IAT (*β* = .24). Participants who explicitly preferred unhealthy food consumed relatively less mineral water than soft drinks. Moreover, participants who implicitly associated themselves more with the healthy food items than with unhealthy food items drank more mineral water and preferred mineral water over soft drinks in the taste comparison task^2^.

## Discussion

It was assumed that the self-regulation of eating behaviour can be understood as a continuum between controlled and automatic processing. Depending on the level of absorption in another task, a shift from more controlled to more automatic processes was supposed. The findings of the present study supported the assumption that within a situation of low distraction (i.e., the food choice task), when controlled processing is possible, nutritional behaviour such as the single choice of fruits versus snacks is predicted by an explicit measure such as self-reported preferences for unhealthy food. Conversely, within a situation that absorbs attention (i.e., high distraction while performing a taste comparison task to cover liquid intake), when automatic aspects of processing are crucial, less controlled nutritional behaviour such as consuming mineral water is predicted by an implicit measure such as the Nutrition IAT. For the difference between mineral water and soft drink intake, however, both the implicit and the explicit measures were relevant. For the IAT as a measure of implicit, automatic processes, the results support the predictions made on the basis of dual-process models (e.g., [1, 2], see also [29]). Automatic processes primarily come into play when an individual is simultaneously engaged in another task that absorbs attention. Controlled processes are especially relevant when a deliberate analysis is possible. Although it may be helpful to draw a clear distinction between these two broad categories of self-regulation, in fact, within situations automatic as well as controlled processing may be involved – although to differing degrees (e.g. see [1], for a discussion of “mixed models”); therefore, controlled, explicit and automatic, implicit self-regulation processes may complement or conflict with each other. For the difference between the mineral water and soft drink intake on the taste comparison task, in addition to the automatic processes, controlled self-regulation processes were also relevant. Although gender served only as a control variable, the additional finding of an especially higher consumption of soft drinks in men as compared to women is in accordance with results from a representative German study [30].

Regarding the choice between fruits and snacks without distraction by another task, the present study supports a study by Karpinski and Hilton [22] in which an IAT failed to predict behavioural food choices. However, our results contrast with the findings of two previous studies [18, 23] that found that an attitude IAT predicted, albeit with a small effect, the behavioural preference for snacks or fruits. It should be noted that the explicit attitude towards snacks was also a significant predictor of behavioural preference [23]. Findings from a meta-analysis of the predictive validity of the IAT [15] showed that implicit measures are capable of predicting both controlled and spontaneous actions. Future studies may continue to systematically test the ability of implicit and explicit measures to predict different food and nutrition behaviours in various contexts in consideration of self-regulation.

The present study’s Nutrition IAT differed from that of previous studies (e.g., [18, 22, 23]) because it integrated a self-concept measure using the categories of self versus other rather than pleasant versus unpleasant. It also used pictures of healthy and unhealthy food items instead of words to increase ecological validity. As usual for IATs (see [12]), the Nutrition IAT showed satisfactory internal consistency. Moreover, it showed predictive and incremental validity for a behavioural measure. With regard to correspondence, it must be noted that the Nutrition IAT with healthy and unhealthy food items predicted the beverage intake. It is quite possible that if the implicit measure (predictor) and the behaviour measure (criterion) had encompassed stimuli from the same domain (i.e., either food or beverages) the prediction might have been stronger (see [15]). Also with regard to the predictive strength of both implicit and explicit measures future studies should been based on the same domain (e.g., beverages). The reliability of the explicit measures was limited for the preferences for healthy and unhealthy food. Thus, it is recommended that future research attempt to improve psychometric properties.

Although Perugini [18] and Richetin et al. [23] comparably operationalised behaviour on the food choice task, the present study’s mineral water and soft drink taste comparison task marks a new behavioural criterion for implicit and explicit predictor studies. Like Friese et al. [21], who manipulated different control resources such as cognitive capacity and self-regulatory resources, the present study implemented as an integral part of the task itself a “taste assessment” to absorb attention and thereby impair controlled processing with regard to the intake of healthy versus unhealthy beverages. To exactly specify the degree of attention absorption and exertion of control in different tasks will be a challenge for future research. For example, how can distraction as an integral part of the task itself as realized in the present study (i.e., with a 5-item taste assessment of four beverages) be compared with an operationalization that relies on additional tasks like digit span procedures [21]? Will it be possible to generalize across different tasks which degree of attention absorption will result in a shift from controlled toward more automatic behaviour?

Healthy eating behaviour is influenced by implicit and explicit processes. Studying the interplay between both processes in sight of social-cognitive models of health behaviour Mai et al. [31] showed that implicit food associations (as measured by a Nutrition IAT) moderate the influence of self-efficacy on eating intentions and behaviour: Participants with more favourable implicit taste associations ate healthier, even if their explicit intentions were low. For effective prevention of chronic diseases, interventions should target not only controlled processes (e.g., [32], for planning interventions) but also automatic processes to promote healthy habits (e.g., [33]) and efficiently change health behaviour [34]. Hollands et al. [35], for example, used an evaluative conditioning procedure that paired images of snack foods with images of potential adverse health consequences (for overviews, see [36, 37]). Evaluative conditioning resulted in changed implicit attitudes (as measured by a Nutrition IAT) with an impact on food choice behaviour.

## Conclusions

This study adds to the literature by showing that nutritional behaviour is influenced by both controlled and automatic processes depending on the available capacity for self-regulation: When distraction was low, food choice was predicted solely by explicit measures (reflecting more controlled processes). On the other hand, when distraction was high, the implicit measure (reflecting more automatic processes) predicted mineral water intake. Thereby in contrast to previous studies distraction was an integral part of the tasks itself. This research strengthens the view that interventions to promote health behaviour may benefit from accounting for controlled as well as automatic processes.

## Footnotes

1 In a different sample of 99 students (77 women and 22 men) aged between 19 and 42 years (*M* = 22.7, *SD* = 3.79), nutrition was assessed with this one item on healthy diet (*M* = 2.62, *SD* = 0.79) and two 8-item scales with healthy and unhealthy food items (i.e., “Please indicate on a scale from 1 = *never* to 5 = *every day* the frequency you eat the following food.” Healthy food consumption: *M* = 26.99, *SD* = 4.81, Cronbach’s *α* = .74, Unhealthy food consumption: *M* = 19.69, *SD* = 4.31, Cronbach’s *α* = .71). The correlation between the healthy and unhealthy food consumption was *r* = −.40, *p* < .001. The one item on healthy diet was positively correlated with healthy food consumption (*r* = .58, *p* < .001) and was negatively correlated with unhealthy food consumption (*r* = −.36, *p* < .001).

2 Hierarchical regression analyses predicting food choice and beverage intake did not change when entering BMI as an additional control variable in variable Block 1 (i.e. gender, age, and BMI).
